# Systematic review of pathways to mental health care in Brazil: narrative synthesis of quantitative and qualitative studies

**DOI:** 10.1186/s13033-018-0237-8

**Published:** 2018-10-31

**Authors:** Carlos Eduardo Amaral, Rosana Onocko-Campos, Pedro Renan Santos de Oliveira, Mariana Barbosa Pereira, Éllen Cristina Ricci, Mayrá Lobato Pequeno, Bruno Emerich, Roseléia Carneiro dos Santos, Graham Thornicroft

**Affiliations:** 1 Department of Collective Health, School of Medical Sciences, University of Campinas, Campinas, Brazil; 2 Department of Psychology, University Centre Unicatólica of Quixadá, Quixadá, Brazil; 3 Unichristus, Fortaleza, Brazil; 4 Department of Community Health, Federal University of Ceará, Fortaleza, Brazil; 5 Centre for Global Mental Health, Institute of Psychiatry, Psychology and Neuroscience, King’s College London, London, UK; 6 Fortaleza, Ceará CEP 60125-001 Brazil

**Keywords:** Pathways to care, Clinical pathways, Systematic review, Public health, Mental health policy

## Abstract

**Background:**

Pathways to care are actions and strategies employed by individuals in order to get help for health-related distress and the related processes of care providers. On several systematic reviews regarding pathways to mental health care (PMHC), studies regarding South American countries were not present. This review synthesizes qualitative and quantitative research about PMHC in Brazil.

**Methods:**

LILACS, MEDLINE and SCIELO databases were searched for papers regarding PMHC in Brazil. The results were organized in pathway stages, based on Goldberg and Huxley’s ‘model of Levels and Filters’ and on Kleinman’s framework of ‘Popular, Folk and Professional health sectors’. Analysis also considered the changes in national mental health policy over time.

**Results:**

25 papers were found, with data ranging from 1989 to 2013. Complex social networks were involved in the initial recognition of MH issues. The preferred points of first contact also varied with the nature and severity of problems. A high proportion of patients is treated in specialized services, including mild cases. There is limited capacity of primary care professionals to identify and treat MH problems, with some improvement from collaborative care in the more recent years. The model for crisis management and acute care remains unclear: scarce evidence was found over the different arrangements used, mostly stressing lack of integration between emergency, hospital and community services and fragile continuity of care.

**Conclusions:**

The performance of primary care and the regulation of acute demands, especially crisis management, are the most critical aspects on PMHC. Although primary care performance seems to be improving, the balanced provision and integration between services for adequate acute and long-term care is yet to be achieved.

**Electronic supplementary material:**

The online version of this article (10.1186/s13033-018-0237-8) contains supplementary material, which is available to authorized users.

## Background

Pathways to care, pathways of care, help-seeking pathways, clinical pathways and therapeutic itineraries are similar (and sometimes overlapping) concepts that refer to the actions and strategies employed by individuals in order to get help for health-related distress [1–5], and/or the related processes of care providers. The actions include attempts to contact other individuals and organizations, both from the formal health system (such as health professionals and services) and other non-statutory agents, such as traditional healers, lay care and social support.

The relevance of clinical presentation, patient choice, system regulation, legitimacy of informal care, and subjective, social and cultural influences on preferred courses of action varies in different definitions of the concept. Vanhaecht et al. [6] identified care pathways as complex interventions, with potential to be used as a model, as a process-oriented improvement strategy or as an *ex post facto* product for research and evaluation. Evans-Lacko et al. [4] argue that the two essential components on different definitions of pathways to mental health care (PMHC) are the types of services and interventions provided; and a timeline assumption of their provision.

Pescosolido [2] distinguished two approaches in the research on help seeking pathways: the “contingency” approach, that describes and correlates service usage with clinical and sociodemographic profiles of patients, and the “process oriented” view, that focuses on social and interpersonal processes that affect help seeking behaviour in the community and in the health system. Cabral et al. [5] identified three main uses for the concept of therapeutic itineraries: the first focused on patient’s perception about illness and how it affects help seeking (patient’s viewpoint); another focused on identifying barriers and gaps in health system accessibility and referral arrangements (system’s viewpoint); and a third integrative approach that considers patient’s actions as part of a socio-economic context that iteratively produces preferred choices and concrete possibilities for both service utilization and informal care (contextual approach).

The characterization of PMHC in the literature can include different ranges of formal and informal care; such pathways might be directed toward specific diagnostic groups or general mental health issues; may be interested in the first contact sought or accessed after feeling distressed, focus on the sources of referral to specialized mental health (MH) care, or trace an exhaustive account of all contacts in retrospective or longitudinal format. The scope of data may be qualitative case studies of an individual’s itinerary, provider, city or country-level patterns of service usage or cross-country comparisons [7–11].

A review of pathways in first episode psychosis (FEP) highlighted that the variety of measures used to report the itineraries compromise direct comparison [12]. This overview found that health professionals are usually the first point of contact, and contact with non-statutory agencies is rare, but in both settings the delay until appropriate care is achieved is considerable. The study also concluded that the cultural determination of pathways of care has not been supported by robust evidence.

Other more recent review of FEP pathways found that physicians were the most common points of first contact, but the most frequent referral source to MH care were emergency services [13]. The review found inconsistent evidence regarding the effects of gender, colour/ethnicity and socio-economic indicators both on the point of first contact and the referral source to specialized MH care. These findings differed both between and within countries. The same inconsistencies also applied to putative associations between longer duration of untreated psychosis (DUP) and place of first contact or referral source.

A review of eight studies using the World Health Organization Encounter Form performed a meta-synthesis on the information of pathways to care for all mental disorders for 23 countries [11]. The paper analysed the time from onset of the MH problem to initial search for care, time until first psychiatric care, self-referral rate to psychiatric services, diagnosis, and main point of access to psychiatric care. However, the results varied greatly depending on context, due to differences in health systems’ design, service provision and cultural values.

There were no studies from Brazil or any other South American country reported in the three reviews cited [11–13], and Mexico and Cuba were the only Latin American countries reporting data, both from the same cross cultural study [8]. This demonstrates a knowledge gap about pathways in Latin America, including Brazil.

### Brazil’s mental health system and polices

Brazil is a higher middle income country, with a GDP per capita of U$8700 [14], a population around 200 million in 2017 [15] and extremely high income inequality, with a 51.3 GINI index [16]. The country is divided in five regions, with the Southeast and South regions being more economically developed than the North and Northeast regions, which in turn also have worse Human Development Indices.

Since 1988 the country has had a universal health system, but with a strong presence of the private sector in health care. Around 25% of the population have private health insurance [17], but there is usually a mixed usage of public and private services [18]. There has been a consistent investment in primary health care in the public sector, with a national coverage rate in 2017 of 74% of the population, but with severe regional disparities [19].

Mental health care is part of the public health system, being primarily community-based, with diminishing presence of psychiatric hospitals. MH policy has suffered major changes in the past four decades. Until the 1980s the MH system was based primarily in psychiatric hospitals, supported by ambulatory care. In the following years, a growing social movement leaded by professionals’ and patient’s associations began to question the ethics and efficacy of the asylum-based model, exposing widespread human rights violations occurring inside psychiatric hospitals [20]. Those actions developed into the Brazilian Psychiatric Reform movement, and in 1987 the first “experimental” community services were created. In 1992 a regulation from the National Ministry of Health made community MH services (*Centros de Atenção Psicossocial*—CAPS) a national policy, and in 2001 a federal law prohibited the creation of new beds in psychiatric hospitals, in order to force a shift in the federal budget towards community services.

In 2006, for the first time the MH budget ratio favoured community care, with 56% of the allocated federal budget, showing a steady increase and reaching 79% in 2013. In 2008 a policy created teams to support primary care staff (*Núcleos de Apoio à Saúde da Família*—NASF) with professionals from different specialties, including MH. From 2011 onwards integrated MH care networks were officially advocated by federal government policies (*Redes de Atenção Psicossocial*), encouraging service integration and variability on the different provider levels (municipal, state and federal levels).

Although CAPS and NASF are currently the main services in the national policy, there are also other services that provide ambulatory care, independently or integrated with psychiatric hospitals. MH crisis are handled primarily in psychiatric hospitals, psychiatric units in general hospitals and in a specific category of 24-h community MH services (type III CAPS). While there are national and local mental health policies, the State has weak regulatory power over the way the services perform, prevailing autonomy in clinical practice. These aspects, along with insufficient service coverage, cause a lot of variability on service availability and singular patterns of MH itineraries in each municipality.

The objective of this review was to explore the characteristics of pathways to mental health care in Brazil, synthesizing evidence from published quantitative and qualitative research. The specific objectives were to articulate the results with different national MH policies adopted over time, and to highlight evidence for each pathway stage.

## Methods

A large variety of study methods and designs addressing PMHC have been used, including qualitative and quantitative studies. Since the PMHCs have considerable sensitivity to context, such broad approach is surely beneficial. However, the traditional systematic review methods would not suffice in analysing and integrating such diversity. Therefore, we used a narrative synthesis, in which a narrative approach is used to integrate evidence, since a statistical approach would be insufficient to handle the results from all relevant sources [21].

The initial search was performed in LILACS, MEDLINE and SCIELO databases, from August to December 2017. Additional file 1 presents details on the search strategies.

The search had no date or language restrictions; included empirical studies using any method or design, either exclusively on MH conditions or at least with separate data and discussion for those conditions, on any age group, performed integrally or partially in Brazil. Studies of any mental disorder were included, encompassing severe and common mental disorders, psychiatric/psychological symptoms, mental suffering, autism and Alzheimer. However, substance abuse, mental paralysis and mental retardation were excluded, since there is a much different array of health services destined specifically for these conditions. Although autism is a developmental disorder, the specialized services for such condition in Brazil are usually child and adolescent community MH services, whereas other developmental disorders, such as mental retardation, are less frequently addressed on those services. Papers that mentioned substance abuse as a co-occurring condition to other MH problems in the inclusion criteria were considered.

Studies of informal or folk care providers were included only if the study authors or participants explicitly matched the nature of the distress with a MH condition described in the inclusion criteria. The screening also considered the following subthemes associated with the concept of PMHC: access; accessibility; globality, continuity of care; and service integration. Additional file 2 details the inclusion and exclusion.

Each study was screened for title and abstract by a pair of independent reviewers (either MLP and BE, or MBP and ECR), and the full text assessment was done by a new pair of reviewers (either PRO and MLP, or CEA and RS). In each stage, any disagreements were settled by independent assessment from a member of the other revision pair.

Quality appraisal was performed using the appropriate Critical Appraisal Skills Program (CASP) checklists, according to each study’s design. One qualitative study was discarded for missing essential information on the description of the research scenario, sampling methods and analysis framework.

### Data analysis

The studies were categorized based on an adaptation of the Levels and Filters Model, originally designed by Goldberg and Huxley [1]. The original model describes the pathway between the community and specialized MH care, with some events being considered filters to move from one level to the next. The first level would be the existing morbity in the community. The second level comprises all the people with MH issues that make contact with a primary care service, while the third level represents the group with conspicuous MH issues. The fourth level are the people referred to specialized MH care, and the last level would be the group actually receiving specialized care [22].

The path from the community until specialized care would have the following series of filters: the decision by the patient to seek medical help; the recognition of a MH problem by the general practitioner (GP); the decision by the GP to provide care or refer to specialized care; the decision by the psychiatrist to provide care. The model highlights that the majority of patients with MH issues is seen in the first level, and a minority, consisting mostly of severe cases, in the last levels [1, 22].

The stages of the model are complementary, although when analysed separately they might shed light in important barriers to mental health care. However, a few modifications on the model were necessary for analytical purposes and adaptation to the Brazilian context. We had to dismiss the notion of primary care as the first logical step in the patient’s decision to consult, and acknowledge instead the decision of where to look for care. This proved useful to analyse the frequent paths with direct access to the community health teams or referrals from hospitals.

Secondly, we considered relevant to include other sources of care besides the formal health system. In this aspect, Kleinman’s framework [23] of a health system comprised of the professional, folk and popular sectors seemed appropriate to organize our data.

Kleinman highlights the cultural and symbolic outlines in treatment and healing experiences of different peoples, stressing how medical interventions are not the only options to be sought and legitimated by people in distress. Instead, the author identifies the coexistence of treatments, rituals and traditions that people submit to, usually seeking more than one type of healing agent. In light of this, Kleinman proposes an explanatory model that integrates those different cultural agents, including not only the formal health professionals, but also the contact with other cultural experts and participation on popular traditions as part of one’s therapeutic itinerary [23].

Finally, on a final addition on our framework, we gave more relevance to the family role in the initial stages of help seeking, in order to address some criticism regarding the Levels and Filter’s model inadequately explaining patients’ behaviour when refusing care [12].

**Fig. 1:**
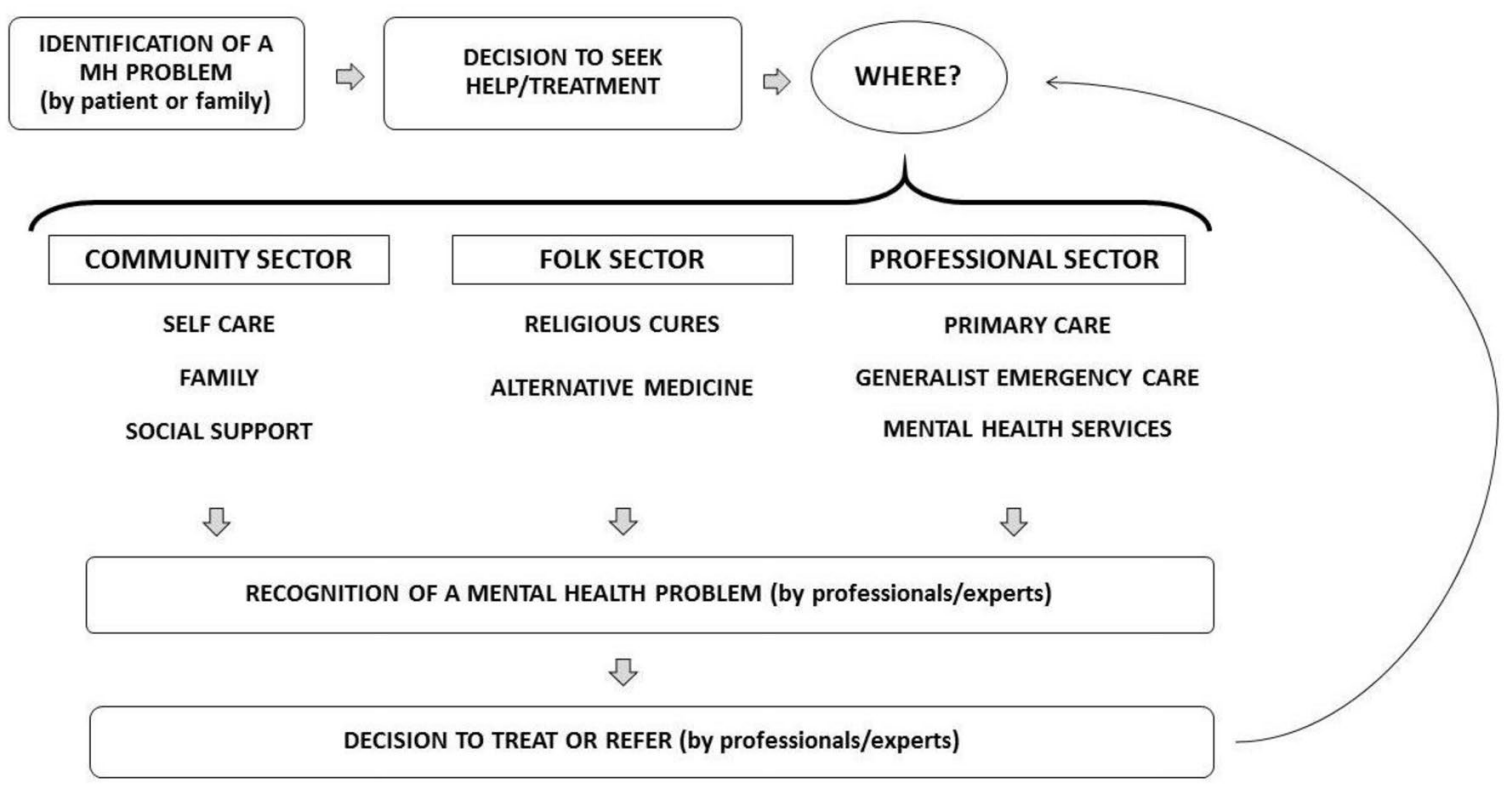
Analytical model

Therefore, we added a stage consisting of the decisions by patients (or their families) to seek care from different agents, which may include, in addition to the formal health system, religious and secular healers, self-help and peer-support strategies, family members and other forms of social support. The overview of the adapted model can be seen in the Fig. 1.

The model allowed relevant data of the reviewed studies to be aggregated and compared within each stage. In addition, challenges and improvements addressed to each filter, as reported by results of individual studies, were synthetized, compared and discussed in the light of the targeted goals of MH policies in place during the period.

Each study provided data for at least one stage, but inclusion in more than one stage was also possible. Data regarding the studies’ dates, methods and scenarios (cities and health services involved, and other places of care, if applicable) were also extracted, as well as targeted diagnosis and age groups, when applicable.

## Results

**Fig. 2:**
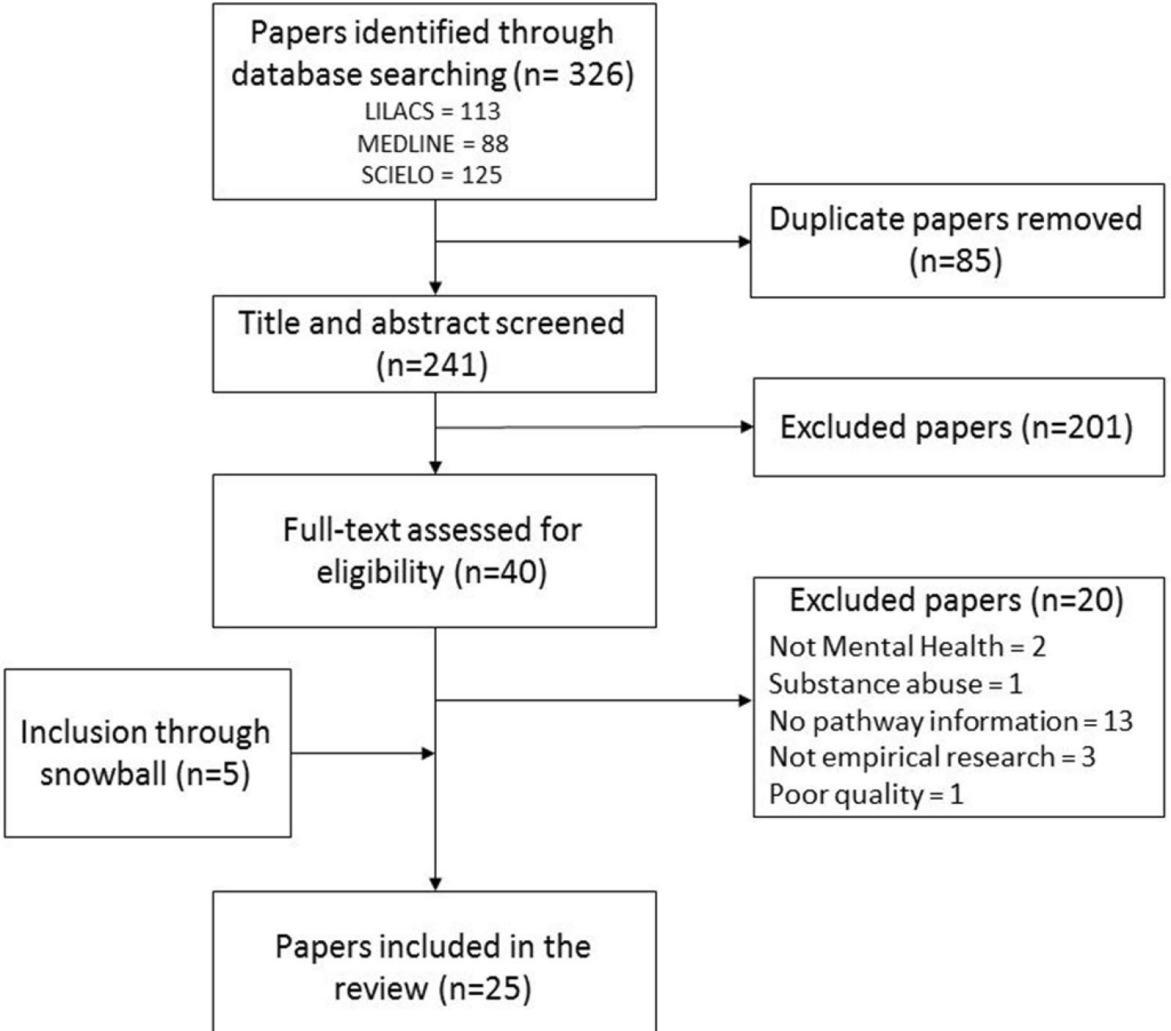
Study selection flowchart

The initial search strategy identified 326 references, which 241 remained after duplicate removal. After title and abstract screening 40 papers were considered in full text assessment, and 21 studies were to be included, but one was discarded for poor quality. The references of these papers were screened for additional studies; experts and authors of studies that appeared to have additional data of interest were contacted. After this process five additional sources were included. See Fig. 2 for the complete flowchart. Authors were also contacted to clarify the period of data collection when the papers did not provide clear information.

The studies ranged widely in terms of design and objectives. Several definitions of pathways to care were found, but there was no usage of any standardized measures. The papers also varied regarding the objectives: most did not have the description of the pathway of care as main goal, but data of interest for one or more stages of our analytical model.

Out of the 25 studies, 9 were quantitative (7 cross-sectional and 2 longitudinal designs), 14 were qualitative and 2 were mixed-methods. All qualitative studies used individual interviews for data collection. As additional sources, 2 studies also used focus groups and 2 performed participant observation.

The papers were published between 1999 and 2017. The studies’ data collection period ranged from 1989 to 2013, and nearly all studies (n = 23) were performed after 2001, when the Psychiatric Reform Law was signed. Over a third of the studies occurred after the Mental Health Integrated Networks policy was approved, and a single study pre-dates the CAPS’ national policy, approved in 1992. The distribution of the studies per year and MH policy period is available in Additional file 3.

In total, evidence from at least 26 cities was provided, with 15 municipalities located in the southeast region, and 11 of those specifically in the state of São Paulo. This concentration is due to one study that targeted 8 different cities in this state. The city of São Paulo, capital of the State of São Paulo, was the most studied municipality (8 papers), which can be explained by the city’s relevance as Brazil’s major economic, scientific and populational centre. A single study from the North region was found, in one city. Some studies did not disclose the city or cities researched. Only 7 papers reported local service provision, in order to provide better context for the findings. Data on research scenarios can be found in Table 1, and the sources of data extraction for each pathway stage can be found in Table 2.

**Table 1:** Studies’ references and researched cities by region and state

Region^a^	State^b^	Number of researched cities	Studies’ references
N	AM	1	[24]
NE	BA	1	[25]
	CE	3	[24, 26, 27]
CW	DF	1	[28]
	GO	1	[24]
	MT	1	[29, 30]
SE	SP	11	[26, 31–39]
	RJ	2	[26, 40, 41]
	MG	2	[24, 42, 43]
S	RS	3	[44, 45]

^a^N: North; NE: Northeast; CW: Center-West; SE: Southwest; S: South

^b^AM: Amazonas; BA: Bahia; CE: Ceará; DF: Distrito Federal; GO: Goiás; MT: Mato Grosso; SP: São Paulo; RJ: Rio de Janeiro; MG: Minas Gerais; RS: Rio Grande do Sul

**Table 2:** Data extraction for each pathway stage

Pathway stage	Recognition of MH problem (by patient or family)	Decision to seek help and choice of care		Recognition of the MH (GP or other non-specialist)	Decision to treat or refer (by GP, other general health services or specialized services)			
Subtheme		Children and adolescents	Adults		Primary care	General health services	Specialized services	Service integration
Articles								
[24]		×						
[25]			×					×
[26]	×	×			×			
[27]				×	×		×	
[28]			×		×			
[29]			×					
[30]				×	×			
[31]	×	×						
[32]	×	×		×				
[33]	×		×	×				
[34]	×	×					×	
[35]		×						
[36]			×					
[37]			×					
[38]				×				×
[39]						×		
[40]	×	×					×	×
[41]	×		×					
[42]	×		×		×			
[43]					×		×	
[44]			×					
[45]			×		×		×	×
[46]	×	×		×				
[47]						×		
[48]							×	
Total	9	8	10	6	7	2	6	4

### Recognition of the mental health problem by the patient or family

A study in 2007 identified that only 59% of parents of children with persistent MH problems considered MH treatment was needed [31]. Two studies on autism highlight the family role in identifying the initial symptoms, especially by the comparison with other children of similar age in order to discriminate delays in speech development and social isolation [32, 46].

Identification by family members is also reported in studies about eating disorders [33, 42], which stress the role of mothers of anorexic or bulimic youths in noticing behaviours such as food selectivity and severe weight loss. However, one of these studies also highlights how people with anorexic or bulimic behaviours do not acknowledge their eating habits or body image perception as inadequate or pathological, showing a conflicting interpretation of their experience in comparison with their families’ and other carers’ [42].

Expectation that schools should take a greater role identifying and treating children and adolescents with mental health issues is reported by parents whose kids have been treated in primary care [26] or child and adolescent community mental health services (CAPSi) [34]. Both studies report that parents consider schools as the most propitious setting to identify early symptoms.

A case study, done in 2012 in Rio de Janeiro, describes an adolescent whose family considered her first mental health crisis as a religious experience [40]. This idea was only dismissed after an encounter with a protestant preacher, who identified it as a health condition. However, only after a second crisis, which led to a psychiatric admittance, that the experience was considered a psychotic episode. Another study done in Rio de Janeiro in 2013, with members of the *Candomble* Afro-Brazilian religion [41], describe a shared sense of belonging, empathy and identification among those that join the religion searching for support for mental distress. The study indicates that although the religion provides care and an explanatory system for the suffering, its members and religious leaders strongly assert the medical perspective as a complementary (and not conflicting) framework of explanation and treatment.

### Decision to seek help and choice of care

#### Children and adolescents

A research done in 1989 in five cities in the São Paulo metropolitan region [35] with children and adolescents with “nervous issues” (*problema dos nervos*), found 141 cases, but only 18% had sought help. From the ones that sought help, 56% (19 cases) contacted a GP, informal help was contacted in 6 (22%) cases, and psychologists in 2 cases. Churches and school were not mentioned as sources of help. Among the cases that did not seek any type of help (109 cases), 61 (56%) considered help was not necessary; 2 referred difficulties to get medical or psychological consultations; and 10 referred “lack of time”, which might also indicate an access barrier to specialized care.

A cohort study in one municipality of São Paulo state [31] identified 124 cases of children (aged 6–13) with MH issues (from a representative sample of 345 children) in 2002. Five years later the sample was reassessed, and 32 cases were identified with persistent MH problems, 16 (50%) had sought treatment, and only 12 (38%) obtained it.

An qualitative inquiry [34] with children and adolescents in treatment at CAPSi in the city of São Paulo revealed that parents face several access barriers during the treatment period, causing treatment abandonment and, hereafter, new searches for help at the same services.

Studies with autistic children highlighted the difficulty to obtain a correct diagnosis, being frequent to visit several health services (primary and specialized care) until the diagnosis is confirmed [32, 46].

A community-level inquiry with children and adolescents with psychiatric disorders [24] identified that 20% of cases with MH issues had used MH providers in the previous 12 months, although the study did not discriminate types of services. The vast majority was seen by psychologists (84.9%), in comparison to 20.9% consulted by psychiatrists and 18.8% by neurologists (several people were seen by more than one type of professional).

Adolescents consulted in primary care centres reported solving problems generally on their own or among peers [26]. They mostly did not see clinics, their family, or other institutions as sources of help. Their parents also did not identify primary care centres as a place to look for MH assistance. Parents felt discouraged by long queues and perceived staff as rushed and uninterested, and generally sought help elsewhere: cardiologists or neurologists (since emotional and behavioural problems could represent problems of the “heart” or “nerves”); friends and neighbours; and religious leaders, such as evangelical pastors and Catholic priests, both for direct advice and referrals. The latter is similar to a case study of an adolescent with severe mental issues [40] that was taken first to a religious temple, subsequently had several intermittent contacts in psychiatric emergency services, until commencing a long-term treatment at a CAPSi.

#### Adults

A research identified help seeking behaviours for three mental health symptoms: anxiousness, insomnia and depressive mood [36]. The vast majority did nothing to address the symptoms, with 6% seeking medical help for anxiousness, 5% for insomnia and 7% for depressive mood. Seeking consultation was less frequent than self-medication for all symptoms (15%, 12% and 10%, respectively). Women had higher percentages than men for both self-medication and consultation.

The preferences of the general public on help seeking for Alzheimer symptoms registered as first choices close family (27%), psychologist (15%), neurologist (13%), self-help group (12%), general practitioner (12%), close friend (9%) and psychiatrist (8%) [29]. Religious leaders, faith healers and pharmacists were rarely selected as a first choice of help. A case study with an elderly woman with a inconclusive Alzheimer diagnosis reported emergency services as the places first sought for help, afterwards MH professionals (several psychiatrists, a neurologist and a psychologist) became the preferred points of contact [44].

In relation to FEP, a cross-sectional research in the city of São Paulo [37] identified that most cases sought help in psychiatric emergency services (74%), and only 26% sought outpatient services. The study also found a very short median DUP (4.1 weeks), attributed to beneficial living arrangements (co-habitation with close relatives) and good emergency service coverage.

Diagnosis and treatment are usually refused by people with eating disorders, therefore the initial contact with the health system is made by the families rather than the patients [42]. In regard of types of professional contacted, out of 21 patients interviewed, 11 looked for medical doctors, 7 went first to psychologists and 2 sought nutritionists. Another study about anorexia from 2012 [33] reports that some mothers went initially to emergency services, after their daughters’ severe malnutrition required hospital admittance.

Although some evidence points to religious figures referring to the formal health system, for most adults from the *Candomblé* religion, the reverse itinerary happened: the formal health system was the first choice for help, and only after the help provided was deemed insufficient that the religious agencies were sought [41]. The religious leaders strongly recommended going through the formal health system beforehand.

Two qualitative papers, with health managers [45] and primary health care workers [28], stress the absence of integrated clinical pathways, usually being left to the patients to control and decide their itinerary in the health system. The studies also reported that different services provide intermittent care, without any provider being able to offer longitudinal follow-up. The health managers describe four main access points for patients seeking MH support: primary care, CAPS, MH ambulatories and hospitals; but since only the hospital has effective response to unscheduled demands, it has the strongest regulatory influence in the clinical pathways [45].

### Recognition of the mental health problem by GP or other general health services

According to a qualitative research with primary care professionals [30], mental health demands are invisible in this level of care: MH issues are frequently not identified nor diagnosed, and the few detected patients have no medical record in the primary care centres. Another study [38] highlights the difficulty for primary care to identify MH demands in homeless people, being dependent on other support teams to approach this group.

Two papers about autism [32, 46] stress that primary care usually is the first contact, but paediatricians and nurses frequently fail to identify the child’s behaviour changes. Difficulty on diagnosis is also reported in one study about eating disorders [33], in which mothers claim that symptoms of anorexia were frequently considered mere “whim” by doctors.

One study [27] reports on the effect of mental health matrix support teams, which allowed primary care workers to better understand mental health disorders. Consequently, identification and treatment of those patients became more frequent, including care to patients with physical problems that had psychological or social determinants or comorbities previously ignored by the health staff.

### Decision to treat or refer: by GP, other general health services and specialized services

#### Primary care

Six qualitative studies identified limited capacity of primary care professionals to treat mental health issues, highlighting referrals to specialized care as the most common practice [26–28, 30, 42, 45]. A qualitative study done in 2005 [26] in three major Brazilian cities reported that primary care professionals tend to offer mental health care only when they perceive referral as impossible due to access barriers. In such cases, they usually performed counselling more based on common sense than on specific training. Another study from 2005, in Cuiabá [30], identified the mental health practices in primary care as either solely medication, or improvised actions. Similarly, in a study form 2008 with primary care staff in the city of Brazlândia-DF [28] the reported mental health interventions were only chatting and guidance, presenting very limited efficacy and frequently needing to refer to psychiatric services.

A study from 2011 done in Fortaleza and Sobral (CE) [27] identified prescription of psychiatric drugs and engaging organized community social resources as mental health actions supported by matrix support teams. A study in 2013 with bulimic and anorexic patients highlights actions from generalist doctors to establish shared case management with psychiatrists, psychologists and nutritionists to provide adequate care [42].

A single study, in Belo Horizonte-MG in 2003, evaluated the de-escalation of care [43], investigating adults with mental disorder that were referred to primary care by a community MH service. The study found that 36% of patients referred after treatment never reached primary care. From the patients actually seen in primary care, even fewer remained in treatment at 9-month follow up (60% of those who reached PHC, and 39% of the initially referred). Referred patients that did not completely cease contact with the specialized MH service after referral had higher odds of successful continuity, both for reaching and maintaining contact with primary care.

#### General health services

A study in São Paulo metropolitan area identified patterns of service use according to different diagnosis (anxiety, mood and substance user disorders), reporting low contact with the folk sector (6% of cases), and increased contact with formal healthcare (24% of cases). From the segment seen in the professional health sector, 70% attended specialized services and 40% generalist services (treatment at both services was possible). Those proportions were similar among each diagnosis within the anxiety and mood disorders group, but not for substance use, which showed an ever higher proportion of specialized service usage (90% vs. 15% of general health services) [47]. Another study from the same research [39] identified that around 40% of cases of MH issues treated in general health services received only medication, while a minority (9%) received a combination of medication and psychotherapy. The proportion of medication-only treatment at specialized services was similar, but the combined treatment was much higher (23%).

#### Specialized services

A study done in 1998 in a community mental health service in Belo Horizonte [43] identified that 17% of their patients came from spontaneous demand, while 30% were referred by primary care, 26% from psychiatric hospitals and 27% from other services.

The “open-doors” policy in the community MH services is highlighted as an ideological principle in a study with CAPS professionals [48], who state that granting initial access to the mental health system through this service is a strategy to strengthen the community-based MH care model and consolidate the change from the previous hospital-based model. However, engaging an actual treatment follow certain protocols to assess severity. Nevertheless, another study [27] stresses how CAPS professionals feel there are excessive inadequate referrals from primary care that overcrowd the CAPS services. One qualitative study with parents of children and adolescents treated in infant and adolescent CAPS [34] points out that, after the initial assessment at these services, several parents gave up due to a long waiting period for the treatment to start.

Although the open-door principle is part of the official CAPS policy, not all services are able to offer unscheduled appointments at a reasonable rate, making the general hospital the first choice for the initial treatment in some cities. This was shown both in a qualitative study with health managers of Santa Maria-RS in 2008 [45] and a case study with an adolescent in the metropolitan region of Rio de Janeiro-RJ in 2012 [40].

#### Service integration

Several studies highlight the difficulty to integrate care between services, once the patients have started treatment. Emergency MH care provided by hospitals is seen as discontinuous and lacking communication with the remaining MH system, which in turn is also seen as fragmented, unable to guarantee continuity of care, and with loose regulation of planned patient flow [25, 38, 40, 45].

## Discussion

Complex social networks were involved in the initial recognition of MH issues and the preferred points of first contact varied with the nature and severity of problems. A high proportion of patients is seen in specialized services, including mild cases. There is limited capacity of primary care professionals to identify and treat MH problems, with some improvement from collaborative care in the more recent years. The model for crisis management remains unclear: scarce evidence was found over the different arrangements used, mostly stressing lack of integration between emergency, hospital and community services and fragile continuity of care.

We identified an important role of social networks supporting the recognition of mental health issues. Besides the self-perception by the person in suffering, other agents such as family, religious agencies and schools were identified as determinants when distinguishing mental health problems.

The studies included in this literature review show that religious figures have an important role not only in offering religious cures and social support, but also in re-directing people to health services. This aspect differs from reports from other middle income countries like Mexico and Cuba, where the search for religious agencies for mental disorders appears scarce [12], but is similar to India, Indonesia and South Africa where religious healers are common in PMHC [11, 49].

The notion of mental suffering originating from magical-religious causes is still common in Brazil; religion and mental health/illness have a profound cultural and historical association [50]. Nowadays the offer of religious cures is not as common anymore in Catholicism, Brazil’s largest denomination. However, it is still quite usual in the other main religions, such as Protestantism, Spiritism, *Candomble* and *Umbanda*. The role of religious agencies is also highly valued in Brazil as a coping strategy, consistent with other Latin-American countries, as well as Latin migrants in other countries [51].

No association has been found between religions and DUP, and different explanatory models (medical and spiritual) seemed to co-exist as complementary in most reports. This differs from evidence from South Africa, where pathways starting from religious healers showed longer DUP and more stages until reaching formal MH care.

Concerning children and adolescent mental health problems, as well as adults with eating or psychotic disorders, family appeared as an important factor on problem identification, decision to seek care and definition of the initial source of help. A considerable short DUP for FEP was found in São Paulo (4.1 weeks), in comparison with international evidence (4–68 weeks, with a within study median of 21.6 weeks) [13]. This appears to be related to the active family role on help-seeking in Brazil, as family support has already been reported as an determining factor to shorten DUP [52, 53]. Studies with Latino populations in the United States have also showed that *familismo*, as a strong Latin-American value, influences individuals to give emphasis on family-level communication, which in turn affects the subsequent help-seeking choices [51].

Nevertheless, there is an apparent conflict of evidence of low frequency of help seeking for children and adolescents with MH issues: 18% of cases with “nervous issues”, reported in 1989; 50% of cases with persistent problems in 2003. The studies address problems with different severities, on distinct periods and cities, but neither report mental health services availability. However, other recent studies in the review reported long waiting periods as access barriers in both primary care and infant and youth community mental health services.

Therefore, between the patients’ decision to seek treatment and the health professional’s decision to treat there might be decisive accessibility issues conditioning these individual decisions, highlighting the importance of a contextual approach [5] when analysing the Brazilian pathways of care. The apparent low frequency of help-seeking for children and adolescent MH issues needs to be contextualized in a scenario of insufficient offer of care [54, 55]. Additionally, hyper-medicalization of common infancy and youth situations, creates an artificial inflation of mental health demands [56, 57].

The choice of the initial help seeking contact seems to vary accordingly to the type of MH demand present. Unspecified symptoms of anxiety, insomnia and depressive mood rarely trigger help seeking on health services, with self-medication being much more common. The studies addressing eating disorders and mental health problems in children and adolescents, report general health services as first contact, while for FEP psychiatric emergency services were the first choice. Studies in different countries also identified divergences in help seeking behaviour according to diagnosis and symptom severity [58, 59].

Identification and treatment of MH problems in primary care and in other general health services are highlighted as challenges in several studies. Neglect and disdain for MH issues, as well as the nature of some interventions, are a cause of concern. The report of actions from primary care teams grounded solely on common sense or exclusively medication-oriented reveal significant limitations. However, a positive impact seems to be happening after investments by the Health Ministry and local health authorities [60, 61]. The more recent studies in the review stress specially the effects of specialized matrix support, from NASF teams and other collaborative care configurations, though studies from after the implementation of NASF teams also report difficulties to adequately address MH in primary care.

The beneficial effects of collaborative care reported include improvements in identification of MH issues, quality of drug prescription, engagement of community resources and shared case management. Other effects reported on the Brazilian literature are increased access to primary care, more adequate referrals to specialized care, support for individual and group psychotherapy, and reduction on stigma [62–65]. International literature points to good outcomes in shared management of depression, mixed results in psychosis and substance abuse and a dearth of studies in anxiety, personality and eating disorders [66].

Continuity of care is a critical issue when referring patients from specialized care back to primary care, as few people seem to reach the primary centres after referral, and fewer adhere to long term follow up. Even in the more recent studies, service integration continues to be a major issue. The definition of the adequate level of case complexity for primary care is also debatable, lacking consensus by NASF professionals and primary care teams [67].

Only a single study, done in the city of Belo Horizonte in 1998, reported the proportion of different referral sources to the CAPS [43]. Therefore, a historical or regional comparison within Brazil was not possible. The found rate of direct access to the CAPS (17%) was similar to the rates to specialized MH care in other middle-income countries (MIC), such as Bangladesh, Bulgaria, India and Indonesia [11]. The proportion of access from GP referrals (30%) was considerable higher than in South Africa (4%) [49], another MIC, but much lower than in Cuba, Spain and the UK (70–85%), middle and high income countries with a strong gatekeeping role by primary care and very low percentage of direct access (0–2.5%) [11].

Although in a stepped care model the access to specialized services should be mediated by primary care, the current mental health system in Brazil was designed with direct access to mental health services, especially to the CAPS. While the gatekeeping role of GPs should ensure high rates of early detection, it can also increase the delay in reaching proper mental health treatment [11]. Additionally, CAPS professionals have been frequently reporting an excessive amount of low-complexity demands at the specialized level, which increases case load and precludes adequate intensity of care for the more severe cases [62–64, 68].

Indeed, the proportion of treated cases that use specialized services is quite high (70%) [39]. Reports on 12-month service use for anxiety, mood, and substance disorders shows that, in high income countries, 37–52% of cases were seen in specialist services, where in middle income countries the proportion varied from 16–54% of the treated cases [69].

Health system organization and professional practice characteristics might be the main predictors for choice and frequency of professional consultation, instead of patient need profiles [70]. Our review shows a lack of service integration in Brazil, in a context of weak regulatory power from health authorities, despite recommendations on an integrated mental health networks policy. Also, the mix of public and private providers in Brazil reinforce health inequities and contribute to increased patient discretion on the pathways of care [18].

General hospitals and emergency services (some of them at psychiatric hospitals) appear to have better accessibility for unscheduled demands, in comparison to most CAPS services. This seems to provide a stronger regulatory power to these services on crisis management and other acute situations, despite being fewer services and having an overall lower volume of patients than the CAPS. Although the integrated care networks policy describes type III CAPS as priority services for managing acute episodes, it also includes both emergency services and primary care centres as planned points of first contact and risk assessment. The model for acute care remains unclear and under pressing debate in Brazil, with claims of emergency services being either central devices for the Psychiatric Reform [71] or reminiscent of the previous asylum-based model [72].

The review also lacked studies focusing on broader aspects of pathways involving psychiatric hospitals. Those services still receive a large volume of patients since the availability of type III CAPS and psychiatric units in general hospitals is utterly insufficient. Scarce evidence over the different models on acute care in Brazil was provided in the reviewed papers.

## Limitations

Although, to the best of our knowledge, this is the first study to review literature on pathways to mental health care in Brazil, as well as the first review to use the progression of national policies over time to compare studies from different periods, this review has some limitations. First, the patterns of PMHC in Brazil seem very dependent of local contextual factors, especially on the provision of different types of services and the possibility of integrated care between them. The lack of comprehensive data on a wider range of settings prevents conclusive reports on nation-wide impacts of the policies. This issue is likely present in other studies of pathways of care in different countries, although the literature tends to generalize evidence from few local sites as national configurations. This might hide important differences in those patterns, which can be a particularly serious problem in a large and unequal country such as Brazil.

A second limitation is that only the qualitative studies included in the review had the analysis of pathways of care as main research objectives, while the quantitative studies provided reports on specific stages of patient itinerary. However, analysing how those individual stages perform in different settings and how they are influenced by policy implementation provided interesting novel results, while simultaneously highlighting gaps in Brazilian literature.

## Conclusions

The performance of primary care and the regulation of acute demands, especially crisis management, seem to be the most critical aspects on the pathways of mental health care in Brazil. Several investments have been done in primary care, whose results have appeared positively in the literature: increased identification, more adequate treatment. However, there is still need for more improvement regarding service coverage, availability of adequate MH treatment and shared management of cases with specialized services. There is a variety of entrance points for the mental health subsystem for different subpopulations, and although primary care influence regulating those flows might have improved, it still has a meagre impact.

There is less evidence available about crisis and acute care, with a dearth in both descriptive reports and impact analysis of the different modes of regulating this demand in Brazil. The challenges and innovations of crisis management in the community are reported in qualitative studies in numerous Brazilian scenarios [73–76], apparently with growing consensus on the necessity of better integration between general hospital and community services [77]. The challenge, however, seems to be how to develop a well-balanced system [78] dealing simultaneously with a low coverage of type III CAPS, insufficient inpatient beds in general hospitals and a suboptimal performance of the MH care network, which might incur on excessive hospitalization demands.

## Additional files

**Additional file 1.** Electronic search strategies.

**Additional file 2.** Inclusion and exclusion criteria.

**Additional file 3.** Studies by year and period of mental health policy.
