# Supplementary material for: Systematic review of pathways to mental health care in Brazil: narrative synthesis of quantitative and qualitative studies
Source: Int J Ment Health Syst. 2018 Oct 31;12:65. doi: 10.1186/s13033-018-0237-8 (PMC6208112; doi:10.1186/s13033-018-0237-8)
Supplement: Supplementary file 1 — Additional file 1. Electronic search strategies. [file 13033_2018_237_MOESM1_ESM.docx]

Additional file 1 – Electronic search strategies

LILACS

tw:(("itinerário terapêutico" OR "itinerário de cuidado" OR "trajetória terapêutica" OR "trajetória de tratamento" OR "trajetória do cuidado" OR "trajetória do paciente" OR "trajetória assistencial" OR "linha de cuidados" OR "comportamento de procura de cuidados de saúde" OR "acesso aos serviços de saúde" OR "critical pathway" OR "clinical pathways" OR "illness itineraries" OR "therapeutic itineraries" OR "health service access" OR "help seeking behavior") AND ("saúde mental" OR psiquiatr* OR psicose OR esquizofrenia OR bipolar* OR depress* OR ansiedade OR "transtorno mental" OR "sofrimento psíquico" OR "mental health" OR "mental disorder" OR "mental suffering" OR psychiat* OR "psychotic disorders" OR "psychosis" OR schizophrenia OR bipolar* OR depress* OR "anxiety") AND (Brasil OR Brazil OR Brésil)) AND (instance:"regional") AND ( db:("LILACS"))

MEDLINE

((“critical pathways” OR “clinical pathways” OR “illness itineraries" OR "therapeutic itineraries” OR “health service access” OR “help seeking behavior”) AND (“mental health" OR "mental disorder" OR "mental suffering" OR psychiat$ OR psychosis OR schizophrenia OR bipolar$ OR depress$ OR anxiety) AND Brazil)

SCIELO

("itinerários terapêuticos" OR "itinerários de cuidado" OR "trajetórias terapêuticas" OR "trajetórias de tratamento" OR "trajetória do cuidado" OR "trajetória do paciente" OR "illness itineraries" OR "therapeutic itineraries" OR "trajetória assistencial" OR "pathways of care" OR "linha de cuidados" OR "comportamento de procura de cuidados de saúde" OR "acesso") AND ("saúde mental" OR "transtorno mental" OR "sofrimento psíquico" OR "sofrimento mental" OR psicose OR psicotic$ OR esquizofren$ OR bipolar$ OR depress$ OR ansiedade OR ansios$)
