# Supplementary material for: Systematic review of pathways to mental health care in Brazil: narrative synthesis of quantitative and qualitative studies
Source: Int J Ment Health Syst. 2018 Oct 31;12:65. doi: 10.1186/s13033-018-0237-8 (PMC6208112; doi:10.1186/s13033-018-0237-8)
Supplement: Supplementary file 2 — Additional file 2. Inclusion and exclusion criteria. [file 13033_2018_237_MOESM2_ESM.docx]

Additional file 2 – Inclusion and exclusion criteria

**Inclusion criteria:**

Any methods and study design.

Any age group.

Mental disorders, mental distress, psychiatric/psychological symptons (including autism and Alzheimer).

Description of pathways between any types of mental health services.

Presence of at least one of the subthemes:

- Access/Acessibility to MH services or professionals (public or private).
- Access/Acessibility to MH interventions on primary care.
- Prevalence, determinants or conditions of seeking mental health services or professionals.
- Prevalence, determinants or conditions of referral to or from mental health services.
- Globality, integrality, continuity or integration (when addressing relations between services or professionals).

**Exclusion criteria:**

Studies outside Brazil.

Other health conditions reported, without separate data on MH.

Substance abuse, brain paralysis, mental retardation or physical deficiencies as theme.

Non-empirical research.
