# Supplementary figures and images for: Systematic review of pathways to mental health care in Brazil: narrative synthesis of quantitative and qualitative studies
Source: Int J Ment Health Syst. 2018 Oct 31;12:65. doi: 10.1186/s13033-018-0237-8 (PMC6208112; doi:10.1186/s13033-018-0237-8)

Additional file 3 - Studies by year and period of mental health policy


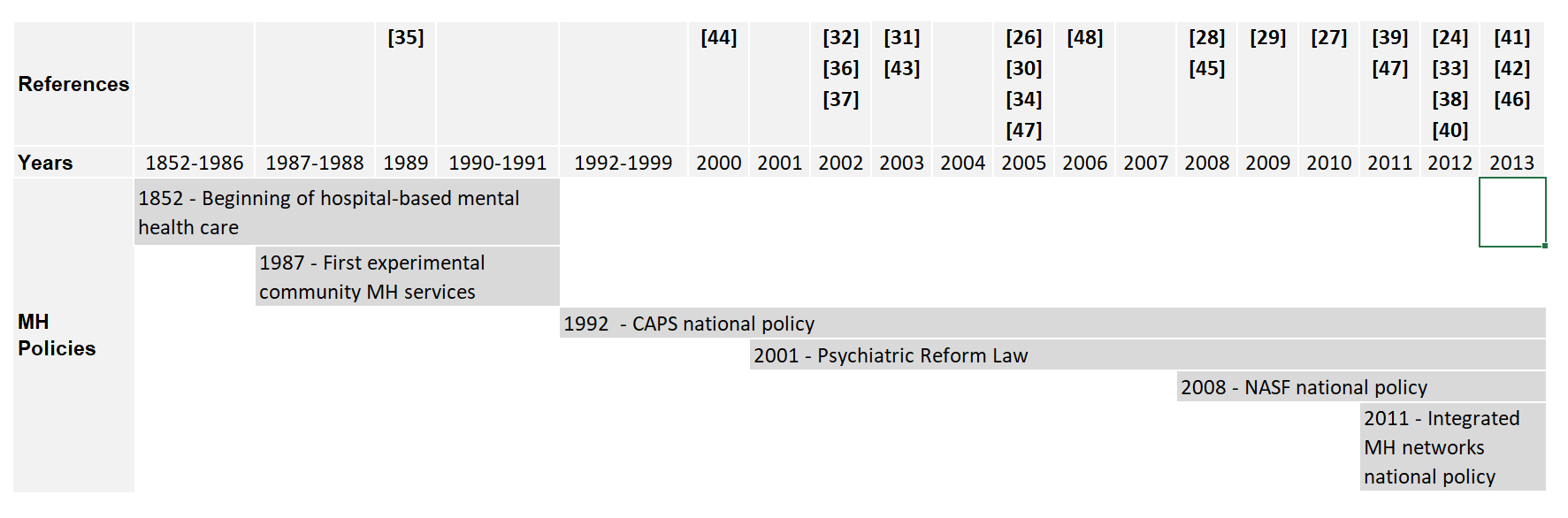

Supplement: Supplementary file 3 — Additional file 3. Studies by year and period of mental health policy. [file 13033_2018_237_MOESM3_ESM.docx]
